# Interleukin-like EMT inducer (ILEI) promotes melanoma invasiveness and is transcriptionally up-regulated by upstream stimulatory factor-1 (USF-1)

**DOI:** 10.1074/jbc.RA118.003616

**Published:** 2018-06-05

**Authors:** Ken Noguchi, Toros A. Dincman, Annamarie C. Dalton, Breege V. Howley, Buckley J. McCall, Bidyut K. Mohanty, Philip H. Howe

**Affiliations:** From the ‡ Department of Biochemistry and Molecular Biology, College of Medicine, and; the § Division of Hematology and Oncology, Medical University of South Carolina, Charleston, South Carolina 29425 and; the ¶ Hollings Cancer Center, Charleston, South Carolina 29425

**Keywords:** epithelial-mesenchymal transition (EMT), melanoma, transcription factor, mRNA, cytokine, FAM3C, ILEI, interleukin-like EMT inducer, phenotype switching, USF-1

## Abstract

Interleukin-like EMT inducer (ILEI, *FAM3C*) is a secreted factor that contributes to the epithelial-to-mesenchymal transition (EMT), a cell-biological process that confers metastatic properties to a tumor cell. However, very little is known about how ILEI is regulated. Here we demonstrate that ILEI is an *in vivo* regulator of melanoma invasiveness and is transcriptionally up-regulated by the upstream stimulatory factor-1 (USF-1), an E-box–binding, basic-helix-loop-helix family transcription factor. shRNA-mediated knockdown of ILEI in melanoma cell lines attenuated lung colonization but not primary tumor formation. We also identified the mechanism underlying ILEI transcriptional regulation, which was through a direct interaction of USF-1 with the ILEI promoter. Of note, stimulation of endogenous USF-1 by UV-mediated activation increased ILEI expression, whereas shRNA-mediated USF-1 knockdown decreased *ILEI* gene transcription. Finally, we report that knocking down USF-1 decreases tumor cell migration. In summary, our work reveals that ILEI contributes to melanoma cell invasiveness *in vivo* without affecting primary tumor growth and is transcriptionally up-regulated by USF-1.

## Introduction

The three most commonly mutated genes in melanoma are *BRAF*, *NRAS*, and *NF1*, all components of the RAS–RAF–MEK^2^–ERK signaling pathway (subsequently referred to as the MEK signaling pathway) (1). Accordingly, MEK signaling plays a major role in melanoma biology by regulating diverse processes such as pigmentation, apoptosis, and senescence (2–9). At a molecular level, MEK signaling affects many transcription factors including the basic-helix-loop-helix leucine zipper (bHLH LZip) transcription factor micropthalmia-associated transcription factor (MITF) (3, 7). MITF binds to E-box motifs (CATGTG) and activates the transcription of the pigment producing gene *PMEL*, which encodes the premelanosome protein, and cell cycle genes such as *CDK2*, which encodes the cyclin-dependent kinase 2 (10, 11). In addition to MITF, MEK signaling affects other bHLH LZip family transcription factors such as upstream stimulatory factor 1 (USF-1) (12, 13). USF-1 binds to E-box motifs (CACGTG) and activates pigmentation genes in response to UV (14, 15).

Interleukin-like EMT inducer (ILEI, *FAM3C*) is a secreted cytokine-like molecule that contributes to the epithelial-to-mesenchymal transition (EMT) (16). EMT is a cell-biological process in which epithelial cells with apical–basal polarity undergo cytoskeletal rearrangement to become motile mesenchymal cells (17). This process is thought to contribute to chemoresistance and metastasis. Although melanoma cells do not undergo a traditional EMT, they utilize a similar process known as phenotype switching. This is a process in which melanoma cells interconvert between a proliferative MITF-high state and an invasive MITF-low state (18–24). Recently, we have described a contribution of ILEI to the invasive MITF-low phenotype *in vitro* (25). Additionally, we showed that phenotype switching between the proliferative MITF-high and invasive MITF-low state modulates ILEI mRNA expression. The molecular regulation of ILEI has focused on post-transcriptional mechanisms including translational regulation by hnRNP-E1–TGF-β and proteolytic processing by plasmin (26–30). However, not much is known about transcriptional regulation of ILEI.

Here, we report that ILEI contributes to melanoma cell line invasiveness *in vivo* and build on our previous finding that vemurafenib (BRAFi) decreases ILEI mRNA expression to characterize the molecular details of ILEI transcriptional regulation by USF-1 (25).

## Results and discussion

### ILEI regulates lung colonization but not primary tumor growth

**Figure 1.**
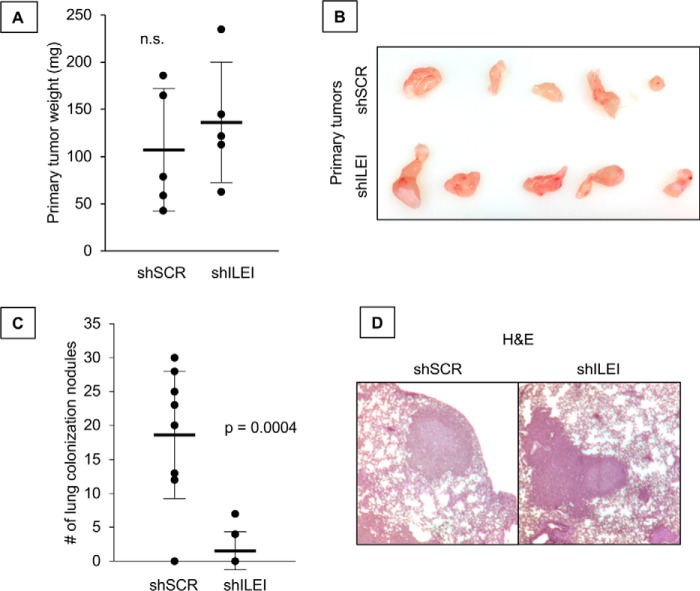
**ILEI regulates lung colonization but not primary tumor growth.** *A,* flank injection of 1205Lu cells expressing shSCR or shILEI. *n* = 5, *bar* indicates mean ± S.D., *n.s*. indicates *p* > 0.05 by Student's *t* test as compared with shSCR. *B,* primary tumors from flank injection experiments. *C,* tail vein injection of 1205Lu cells expressing shSCR or shILEI. *n* = 9 for shSCR and *n* = 7 for shILEI, *bar* indicates mean ± S.D., *p* value indicated by Student's *t* test as compared with shSCR. *D,* representative images of H&E-stained lung nodules from either shSCR or shILEI.

Previously we found that knockdown of ILEI contributes to melanoma invasiveness *in vitro*, whereas ILEI had no effect on proliferation (25). Here we used the same ILEI-modulated cell lines and conducted *in vivo* flank and tail vein injections to measure primary tumor formation or lung colonization, respectively. In the flank injection experiment we found no significant difference in primary tumor growth (Fig. 1, *A* and *B*), but we observed in the tail vein injection experiment that ILEI knockdown significantly attenuated lung colonization (Fig. 1*C*). We did not observe a difference in the size of the lung colonies formed by either shSCR or shILEI cell lines, suggesting that the ability to invade the lung was affected rather than the ability to grow metastatic colonies in the lung (Fig. 1*D*). Based on these results we conclude that ILEI specifically regulates melanoma invasiveness *in vivo* without affecting primary tumor growth.

### Vemurafenib inhibits ILEI expression

To date there are three known mechanisms of ILEI regulation: the first is translational regulation of ILEI by TGF-β–AKT2–hnRNP-E1, the second is degradation of ILEI by the ubiquitin/proteasome system, and the third is an autophagy-mediated increase in ILEI protein expression (16, 25, 26, 30–33). Nothing has been reported about the mechanistic basis of ILEI transcription. None of the previously established mechanisms appears to be responsible for ILEI regulation by vemurafenib (25). Herein, we sought to determine the mechanism of ILEI transcriptional regulation.

**Figure 2.**
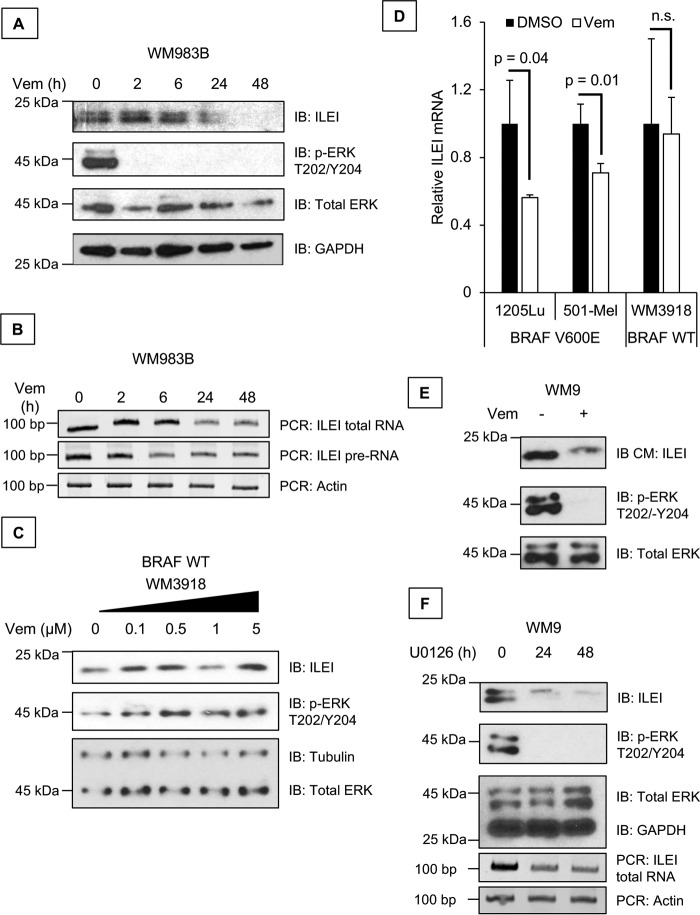
**Vemurafenib inhibits ILEI expression.** *A,* immunoblot (*IB*) analysis of ILEI, *p-*ERK, ERK, and GAPDH levels in WM983B melanoma cell lines treated for 0 to 48 h with vemurafenib (1 μm). *B,* RT-PCR analysis of ILEI, ILEI intron indicates that PCR primers targeted the pre-RNA but not mRNA of the *ILEI* gene, and Actin levels in WM983B melanoma cell lines treated for 0 to 48 h with vemurafenib (1 μm). *C,* immunoblot analysis of ILEI, *p-*ERK, ERK, and Tubulin levels in WM3918 melanoma cell lines treated for 24 h with vemurafenib (0–5 μm). *D,* bar diagram showing quantitative RT-PCR analysis of ILEI levels in 501-Mel, 1205Lu, or WM3918 melanoma cell lines treated for 24 h with vemurafenib (1 μm). *n* = 3, mean ± S.D., *p* value indicated by Student's *t* test as compared with vehicle treatment, transcript values are normalized to GAPDH. *E,* immunoblot analysis of ILEI, *p-*ERK, and ERK levels in WM9 melanoma cell lines treated for 0 or 24 h with vemurafenib (1 μm). *IB CM* indicates that the serum-free medium condition for 24 h during vemurafenib treatment was harvested and TCA precipitated for immunoblot analysis. *F,* immunoblot and RT-PCR analysis of ILEI, *p-*ERK, ERK, GAPDH, and actin levels in WM9 melanoma cell lines treated for 0 to 48 h with U0126 (MEKi, 10 μm).

First, we treated WM983B melanoma cell lines with vemurafenib, an inhibitor of V600E BRAF, and observed that ILEI protein and mRNA expression decreased at 24 h (Fig. 2, *A* and *B*) (25). We also conducted RT-PCR using primers targeting an intronic sequence of ILEI to amplify ILEI pre-RNA (Fig. 2*B*). If ILEI mRNA levels are regulated post-transcriptionally by miRNAs, we expect that ILEI pre-RNA should not be affected by vemurafenib. However, if ILEI mRNA levels are regulated transcriptionally, we expect that ILEI pre-RNA should go down upon vemurafenib treatment. We saw that vemurafenib treatment decreased both ILEI total RNA and pre-RNA, suggesting that vemurafenib affected ILEI transcription (Fig. 2*B*). To confirm that these findings were not due to off-target effects of vemurafenib we conducted a control experiment in which we used the *BRAF* WT WM3918 melanoma cells. Vemurafenib is a specific inhibitor of mutant BRAF, and it does not affect WT BRAF (34). Therefore, we treated *BRAF* WT WM3918 melanoma cells with vemurafenib, which should retain all the nonspecific effects of vemurafenib without the BRAF-specific effects. We found that vemurafenib did not affect ILEI protein expression or ERK phosphorylation in *BRAF* WT WM3918 melanoma cells (Fig. 2*C*). We confirmed these findings by a quantitative method using real-time qPCR and found that vemurafenib decreased ILEI expression in *BRAF* mutant 501-Mel and 1205Lu cells but not in *BRAF* WT WM3918 cells (Fig. 2*D*). Considering that ILEI is a secreted cytokine, we wanted to confirm the physiological relevance of vemurafenib-mediated ILEI inhibition by conducting immunoblot analysis of the conditioned medium. These results confirmed that vemurafenib decreased secreted ILEI levels (Fig. 2*E*). Finally, to further determine whether this effect is specific for the MEK pathway we used a second inhibitor of the MEK pathway (U0126, MEKi) and observed decreased ILEI expression by U0126 (Fig. 2*F*). Based on these results we conclude that vemurafenib inhibits ILEI mRNA expression, and that this effect depends on the presence of oncogenic *BRAF* V600E mutation.

### Vemurafenib inhibits ILEI at the transcriptional level

**Figure 3.**
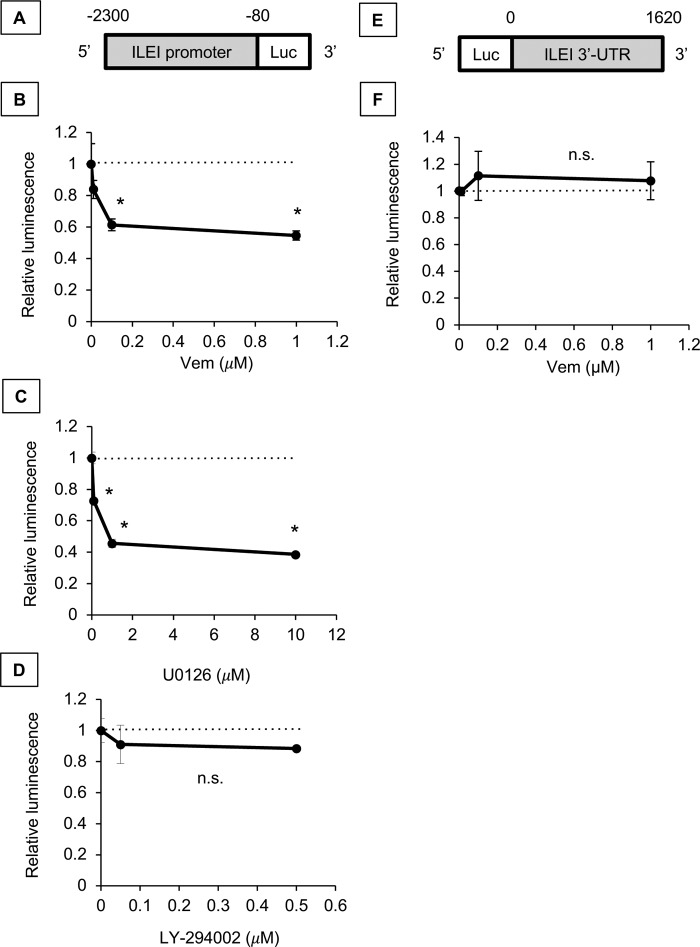
**Vemurafenib inhibits ILEI expression at the transcriptional level.** *A,* design of ILEI promoter reporter construct. The promoter reporter construct spans 2300 bp upstream of the TSS to 80 bp upstream of the TSS. *B,* luciferase assay of ILEI promoter reporter construct in 501-Mel melanoma cell lines treated for 24 h with vemurafenib (BRAFi, indicated concentration). *n* = 3, mean ± S.D., * indicates *p* < 0.01 by Student's *t* test as compared with vehicle treatment. *C,* luciferase assay of ILEI promoter reporter construct in 501-Mel melanoma cell lines treated for 24 h with U0126 (MEKi, indicated concentration). *n* = 3, mean ± S.D., * indicates *p* < 0.01 by Student's *t* test as compared with vehicle treatment. *D,* luciferase assay of ILEI promoter reporter construct in 501-Mel melanoma cell lines treated for 24 h with U0126 (MEKi, indicated concentration). *n* = 3, mean ± S.D., * indicates *p* < 0.01 by Student's *t* test as compared with vehicle treatment. *E,* design of ILEI 3′-UTR reporter construct. The 3′-UTR reporter construct spans from the end of the coding sequence to 1620 bp downstream. *F,* luciferase assay of the ILEI 3′-UTR reporter construct in 501-Mel melanoma cell lines treated for 24 h with vemurafenib (indicated concentration). *n* = 3, mean ± S.D., *n.s*. indicates *p* > 0.05 by Student's *t* test as compared with vehicle treatment.

We cloned the ILEI promoter from 2,300 bp upstream of the transcription start site (TSS) to 80 bp upstream of the TSS into pBV-Luc (Fig. 3*A*) (35). We transfected 501-Mel cells with the ILEI promoter luciferase reporter construct along with a control *Renilla* construct and treated the cells with vemurafenib (Fig. 3*B*). We found that vemurafenib decreased the ILEI promoter activity in a dose-dependent manner. As further controls we tested the ILEI promoter construct with the MEK inhibitor U0126 and found that U0126 decreased promoter activity (Fig. 3*C*). However, when we tested the ILEI promoter construct with the PI3K inhibitor LY-294002, we found that promoter activity was not affected (Fig. 3*D*). Additionally, we cloned the ILEI 3′-UTR from the stop codon to the end of the mRNA at 1,620 bp into pmirGLO Dual Luciferase (Fig. 3*E*). We transfected 501-Mel cells with the ILEI 3′-UTR construct and treated the cells with vemurafenib (Fig. 3*F*). We found that vemurafenib did not affect the ILEI 3′-UTR construct. From these experiments we concluded that vemurafenib regulates ILEI mRNA at the transcriptional level.

**Figure 4.**
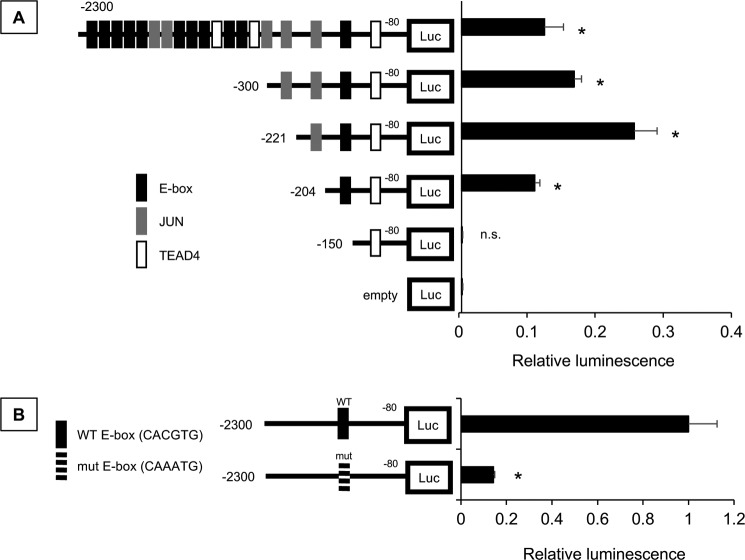
**Proximal E-box contributes ILEI promoter activity.** *A,* luciferase assay of various length ILEI promoter reporter constructs in 501-Mel melanoma cell lines. *n* = 3, mean ± S.D., *p* value indicated by Student's *t* test as compared with the empty promoterless reporter. *B,* luciferase assay of WT and E-box mutant (CACGTG to CAAATG) ILEI promoter reporter constructs (−2300/−80) in 501-Mel melanoma cell lines. *Black bars* indicate WT and *white bars* E-box mutant. *n* = 3, mean ± S.D., *p* value was indicated by Student's *t* test comparing E-box mutant to WT.

Because vemurafenib regulates ILEI transcription, we hypothesized that a *cis* element in the ILEI promoter region regulates this phenomenon. We analyzed a 2,300-bp sequence at the ILEI promoter from the TSS for various transcription factor motifs using the JASPAR database (36). Previously we had found that ILEI is highly expressed in MITF-low invasive melanoma cell lines, so we focused our transcription factor search on regulators of the MITF-low invasive state (E-box (ZEB1/2), JUN, and TEAD4) (20, 21, 23, 37–39). We found 11 putative E-box, 5 JUN, and 3 TEAD4 sites in the ILEI promoter, and generated successive 5′-deletions of the ILEI promoter reporter (Fig. 4*A*). We transfected 501-Mel cells with these constructs and observed that the truncation from −204 to −150 reduced the luciferase activity to empty promoter control levels. There was an E-box motif in this region, so we next wanted to know if this E-box was specifically important for ILEI promoter activity or if any E-box would suffice. Thus, we used our longest ILEI promoter reporter (−2300 to −80) and specifically mutated the E-box consensus site at −163 from CACGTG to CAAATG. Again, we observed a marked inhibition in luciferase activity (Fig. 4*B*). From these experiments we concluded that an E-box 163 bp upstream of the ILEI TSS is critical to basal ILEI promoter activity.

**Figure 5.**
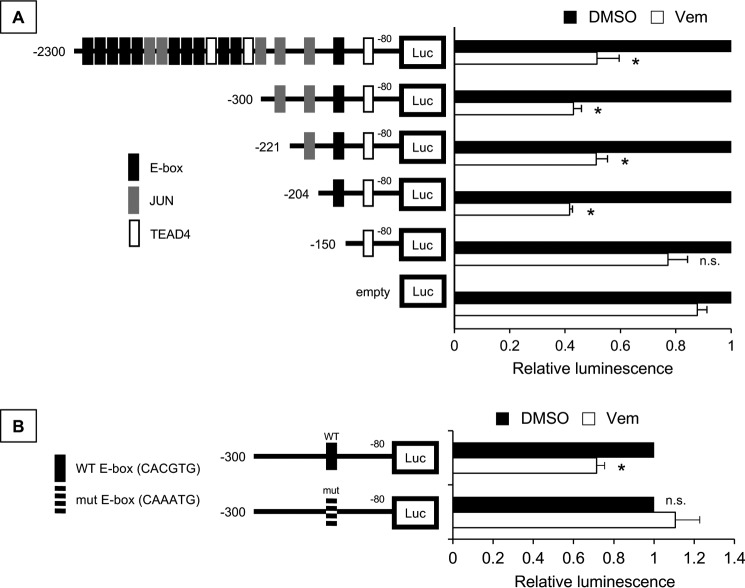
**Proximal E-box contributes ILEI promoter vemurafenib responsiveness.** *A,* luciferase assay of various length ILEI promoter reporter constructs in 501-Mel melanoma cell lines. *Black bars* indicate control DMSO treatment and *white bars* indicate 24 h treatment with vemurafenib (1 μm). Values are normalized to DMSO treatment of the particular reporter construct. *n* = 3, mean ± S.D., *p* value was indicated by Student's *t* test as compared with vemurafenib treatment of the empty promoterless reporter. *B,* luciferase assay of WT and E-box mutant (CACGTG to CAAATG) ILEI promoter reporter constructs (−300/−80) in 501-Mel melanoma cell lines. *Black bars* indicate control DMSO treatment and *white bars* indicate 24 h treatment with vemurafenib (1 μm). *n* = 3, mean ± S.D., *p* value was indicated by Student's *t* test comparing vemurafenib to DMSO treatment.

Next, we wanted to know the role of this E-box motif in the vemurafenib-mediated regulation of ILEI expression. We transfected 501-Mel cells with the ILEI promoter truncation constructs and treated the cells with vemurafenib (Fig. 5*A*). We found that truncation of bp −204 to −150, which includes an E-box motif, eliminated ILEI promoter vemurafenib responsiveness. The −204 to −150 region of the ILEI promoter contains 48 bp in addition to the E-box, so we mutated the E-box in the −300/−80 construct. We sequenced the construct and analyzed the sequence in the JASPAR database to find that our mutations abolished all putative binding sites in this region (data not shown). We conducted luciferase assays and observed that mutation of the E-box similarly eliminated vemurafenib responsiveness (Fig. 5*B*). From these experiments we concluded that vemurafenib inhibits ILEI at the transcriptional level through an E-box 163 bp upstream of the ILEI TSS.

### USF-1 directly regulates ILEI transcription

**Figure 6.**
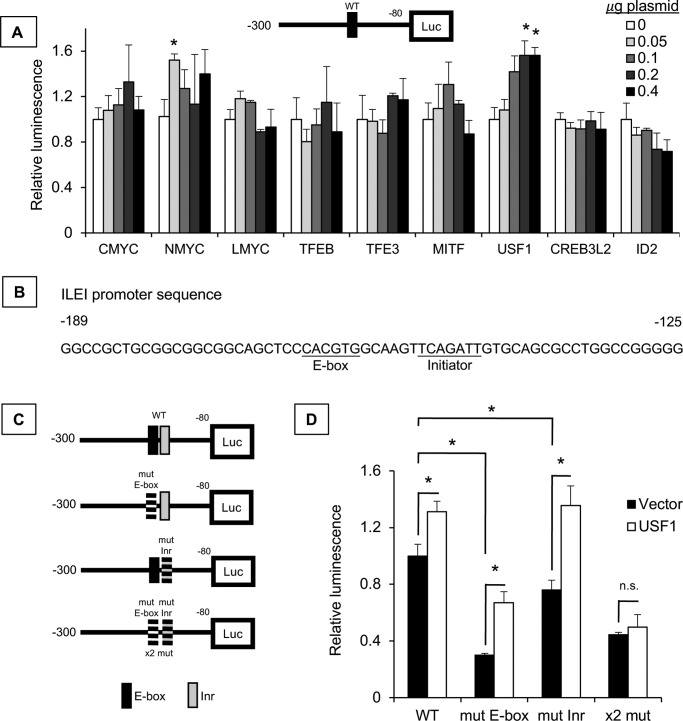
**USF-1 directly regulates ILEI transcription.** *A,* luciferase assay of the ILEI promoter reporter construct (−300/−80) in 501-Mel melanoma cell lines. 0, 0.05, 0.1, 0.2, and 0.4 μg of experimental vector and a corresponding 0.4, 0.2, 0.15, 0.1, or 0 μg of empty vector were transfected for 24 h. The increased darkness of the bars indicates increased experimental vector. Luminescence is normalized to vector control for each transcription factor. *n* = 3, mean ± S.D., * indicates *p* < 0.01 by Student's *t* test as compared with vector transfection. *B,* ILEI promoter sequence from −189 to −125 highlighting E-box and initiator (Inr) motifs. *C,* various ILEI promoter luciferase reporter constructs either WT or mutant for the E-box or the Inr. *D,* luciferase assay of ILEI promoter reporter constructs in 501-Mel melanoma cell lines. *Black bars* indicate vector overexpression and *white bars* indicate USF-1 overexpression. *n* = 3, mean ± S.D., *p* value indicated by Student's *t* test as compared with vector.

Considering the importance of the E-box motif to ILEI expression, we wanted to identify the trans-acting factor regulating this effect. To this end, we overexpressed different transcription factors known to bind E-box motifs and found that USF-1 induced ILEI promoter activity, but not c-MYC, N-MYC, l-MYC, TFEB, TFE3, MITF, CREB3L2, and ID2 (Fig. 6*A*). USF-1 activates transcription through the E-box motif, but also through a pyrimidine-rich initiator sequence (Inr) (40). The ILEI promoter sequence has an Inr sequence 6 bp downstream of the E-box (Fig. 6*B*). Thus, we generated Inr mutant ILEI promoter constructs in addition to our E-box mutants to see if USF-1 regulated ILEI promoter activity directly through the USF-1–binding sites (Fig. 6*C*). We observed that USF-1 induced ILEI promoter activity in the WT promoter, the E-box mutant promoter, the initiator mutant promoter, but not the E-box and Inr double mutant promoter (Fig. 6*D*). From these experiments we concluded that USF-1 directly regulates ILEI promoter activity.

**Figure 7.**
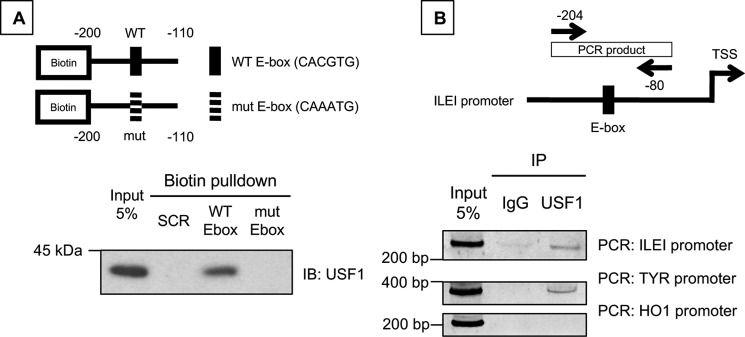
**USF-1 interacts with the ILEI promoter sequence.** *A,* 5′-biotin–tagged ILEI promoter oligonucleotide constructs with WT or mutant E-box. Biotin pulldown analysis of nuclear extracts from 501-Mel melanoma cells, followed by immunoblot for USF-1. *B,* PCR primers flanking the ILEI promoter E-box used in ChIP analysis. ChIP analysis of 501-Mel melanoma cell lines immunoprecipitated with control IgG or USF-1 antibody. PCR analysis conducted with primers targeting *FAM3C, TYR,* or *HO1* promoter.

We wanted to further prove a direct role of USF-1 in ILEI regulation so we pursued a potential interaction between the ILEI promoter and USF-1. We conducted streptavidin pulldown experiments using 5′-biotin–tagged ILEI promoter constructs either WT or mutant for the E-box (200 to 110 bp upstream of ILEI TSS, Fig. 7*A*) and 501-Mel melanoma cell nuclear extract. We found that USF-1 can be isolated from cell nuclear extract using the WT ILEI promoter, but not the E-box mutant ILEI promoter, or a control random 60-bp oligonucleotide (SCR, Fig. 7*A*).

Interestingly, the E-box mutation was sufficient to abolish binding by the streptavidin pulldown experiment, but the E-box mutation was not sufficient to abolish USF-1-mediated induction of the ILEI promoter reporter (Fig. 6*D*). We speculate either that 1) USF-1 still binds the ILEI promoter at levels that are undetectable by our streptavidin pulldown assay, or 2) endogenous USF-1 binds only to the E-box but overexpressed exogenous USF-1 provides excess USF-1 to bind both the E-box and the Inr. This is supported by the finding that basal ILEI promoter activity is affected more by the mutation of the E-box than the Inr (Fig. 6*D*). This is also consistent with previously published findings that show the E-box is the high-affinity USF-1–binding site, whereas the Inr is the low-affinity binding site (41).

To confirm that the interaction between USF-1 and the ILEI promoter was physiologically relevant, we conducted ChIP using USF-1 antibody and PCR primers flanking the ILEI promoter E-box (Fig. 7*B*). We confirmed the efficacy of our assay by showing that USF-1 IP can detect a segment of the *TYR* promoter (15), and we detected an interaction between USF-1 and the ILEI promoter (Fig. 7*B*). However, we found that a segment of the HO1 promoter, which is a USF-1 interaction reported in epithelial cells, was not detected in our melanoma cells (42). From these experiments we concluded that USF-1 directly interacts with the ILEI promoter and this interaction is E-box dependent.

### USF-1 in vemurafenib-regulated ILEI expression

Thus far we have shown that vemurafenib inhibits ILEI transcription through an E-box motif, and that USF-1 directly regulates ILEI transcription through the same E-box motif. Next, we wanted to understand the role of USF-1 in vemurafenib-mediated regulation of ILEI transcription.

**Figure 8.**
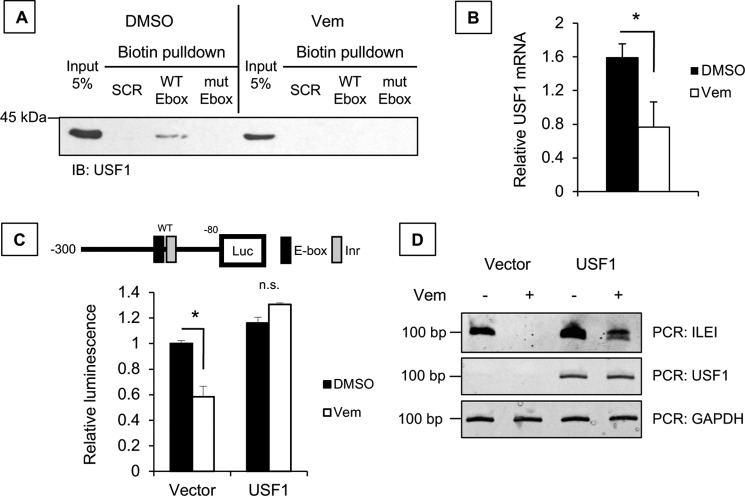
**USF-1 in vemurafenib-regulated ILEI expression.** *A,* biotin pulldown analysis of nuclear extracts from DMSO or vemurafenib-treated 501-Mel melanoma cells, followed by immunoblot for USF-1. *B,* qPCR analysis of USF-1 in Sk-Mel-28 melanoma cells treated with DMSO or vemurafenib (24 h, 0 μm). *C,* luciferase assay of the WT ILEI promoter reporter construct (−300/−80) in 501-Mel melanoma cell lines with vector or USF-1 overexpression and DMSO or vemurafenib treatment (1 μm). *Black bars* indicate DMSO and *white bars* indicate vemurafenib. *n* = 3, mean ± S.D., *p* value indicated by Student's *t* test as compared with DMSO treatment. *D,* PCR analysis of ILEI, USF-1, and GAPDH in 501-Mel melanoma cell lines overexpressing vector or USF-1 and treated with vemurafenib (24 h, 5 μm).

For this we employed the streptavidin pulldown assay using 5′-biotin–tagged ILEI promoter constructs WT or E-box mutant in cells treated with vemurafenib. First, we validated the binding of USF-1 to the ILEI promoter (DMSO condition) and showed that vemurafenib treatment abolished USF-1 interaction with the ILEI promoter (Fig. 8*A*). Importantly, we noticed by comparing the input lanes that USF-1 expression is decreased by vemurafenib treatment. We used qPCR to confirm that vemurafenib represses the mRNA levels of USF-1 (Fig. 8*B*).

If vemurafenib inhibits ILEI expression by repressing USF-1, overexpression of USF-1 should rescue vemurafenib-mediated inhibition of ILEI. Thus, we overexpressed USF-1, treated with vemurafenib, and tested ILEI promoter activity. We found vemurafenib regulation of ILEI expression in vector–overexpressing cells, but not in USF-1–overexpressing cells (Fig. 8*C*). In fact, we observed that vemurafenib treatment actually induced ILEI promoter activity in the context of USF-1 overexpression. We propose the following explanation: in the absence of exogenous USF-1, vemurafenib decreases USF-1 expression to inhibit ILEI transcription (Fig. 8*B*), but in the presence of exogenous USF-1, vemurafenib activates p38 kinase to activate USF-1 and counterintuitively activate ILEI transcription (15, 20).

Finally, we confirmed these results with the endogenous ILEI by overexpressing USF-1, treating with vemurafenib, and conducting PCR analysis. Although the rescue was not complete, we observed that USF-1 overexpression rescues vemurafenib inhibition of ILEI (Fig. 8*D*). From these experiments we concluded that the mechanism of vemurafenib-mediated inhibition of ILEI is through down-regulation of USF-1 mRNA.

### Role of endogenous USF-1 in ILEI expression

**Figure 9.**
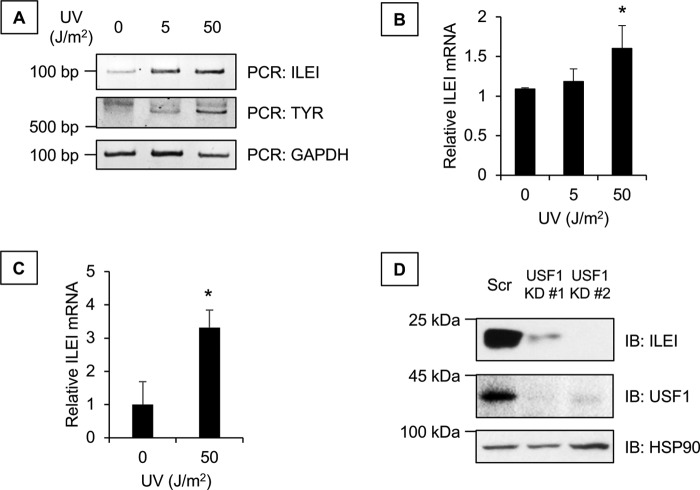
**Effect of endogenous USF-1 on ILEI expression.** *A,* PCR analysis for ILEI, tyrosinase, or GAPDH of 501-Mel melanoma cells 24 h after treatment with 0, 5, or 50 J/m^2^ of UV. *B,* qPCR analysis of ILEI in 501-Mel melanoma cells 24 h after treatment with 0, 5, or 50 J/m^2^ of UV. *n* = 3, mean ± S.D., * indicates *p* < 0.05 compared with 0 UV by Student's *t* test. *C,* qPCR analysis of ILEI in HMLE human mammary epithelial cells 24 h after treatment with 0 or 50 J/m^2^ of UV. *n* = 3, mean ± S.D., * indicates *p* < 0.05 compared with 0 UV by Student's *t* test. *D,* immunoblot analysis of ILEI, USF-1, or HSP90 in 501-Mel melanoma cells transduced with lentivirus containing scrambled shRNA or two different sequences targeting *USF1*.

We wanted to assess if endogenous USF-1 had a role in ILEI expression. USF-1 is known to be regulated by stress-mediated p38 MAPK. Mechanistically, p38 phosphorylates USF-1 on threonine 153, which activates its transcriptional activity for target genes, including *TYR* (15). Thus, we used UV treatment as a model of endogenous USF-1 activation and observed that UV increased ILEI mRNA both by RT- and qPCR (Fig. 9, *A* and *B*). Furthermore, we wanted to know if UV–USF-1–ILEI regulation was intact in nonmelanoma cell lines. We used HMLE human mammary epithelial cells and observed that UV increased ILEI mRNA by qPCR (Fig. 9*C*). In addition to activation of endogenous USF-1, we wanted to test the effect of inhibiting endogenous USF-1. We used two different shRNA molecules specific for USF1 and observed that knockdown of endogenous USF-1 inhibits ILEI expression (Fig. 9*D*). From these experiments we concluded that endogenous USF-1 regulates ILEI expression.

**Figure 10.**
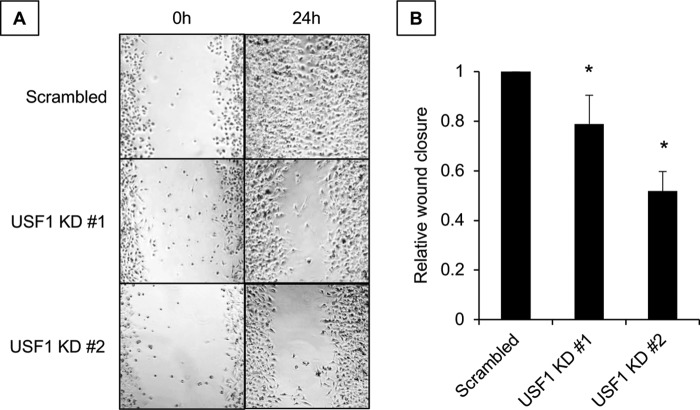
**Biological role of the USF-1–ILEI axis in melanoma cells.** *A,* representative images of wound-healing assays of 501-Mel melanoma cells transduced with lentivirus containing scrambled shRNA or two different sequences targeting *USF1. B,* quantification of *A* using ImageJ software. *n* = 3, mean ± S.E., and * indicates *p* < 0.05 compared with scrambled by unpaired Student's *t* test.

Finally, we wanted to know if USF-1 regulation of ILEI had any biological significance. Given the importance of ILEI to the invasive melanoma phenotype (Fig. 1), we tested the contribution of USF-1 to wound healing. We observed that USF-1 knockdown in 501-Mel melanoma cells attenuates wound healing (Fig. 10, *A* and *B*). From these experiments we concluded that USF-1 regulates migration in melanoma cell lines.

**Figure 11.**
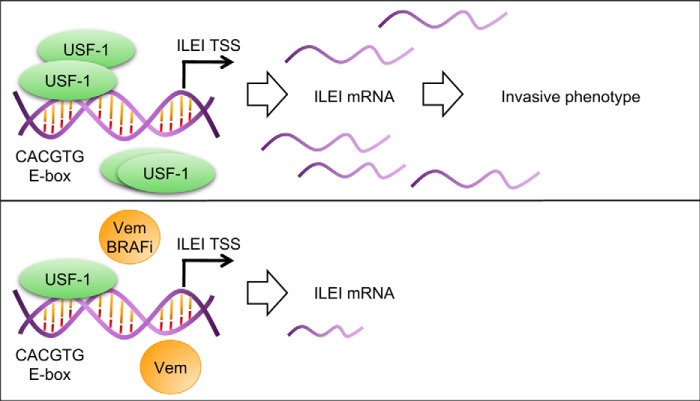
**Model of USF-1 regulation of ILEI.** A model depicting basal *versus* vemurafenib-treated conditions is shown. *BRAFi,* B-Raf kinase inhibitor. *ILEI*: interleukin-like EMT inducer, *FAM3C. Vem,* vemurafenib.

In summary, we have described the following novel findings: ILEI regulates melanoma invasiveness *in vivo*, vemurafenib inhibits ILEI at the transcriptional level through a specific E-box sequence, USF-1 directly interacts with this E-box in the ILEI promoter and that this interaction is abolished upon vemurafenib treatment, and finally that endogenous USF-1 contributes to ILEI expression and the invasive melanoma phenotype. Through these findings we have established a novel regulatory mechanism for ILEI expression by USF-1, and based on this previously unknown context for ILEI expression we speculate on the following new biological functions for USF-1 and ILEI (Fig. 11).

Although ILEI has been thoroughly described as a tumor autonomous regulator of EMT and invasion, the role of ILEI in paracrine signaling is still unclear (25, 28, 29, 33, 43–45). Our characterization of the novel vemurafenib–USF-1–ILEI regulatory axis has implications for paracrine ILEI signaling. Given the impact of vemurafenib on *BRAF* mutant melanoma, the mechanisms of vemurafenib-mediated tumor killing have been thoroughly characterized. For instance, mouse models have shown that vemurafenib induces apoptosis of tumor suppressive regulatory T cells while simultaneously promoting CD8 T cell-mediated killing of tumor cells (46). Other models have shown that vemurafenib increases the recruitment of macrophages to *BRAF* mutant melanomas (47). Combining our finding that vemurafenib inhibits USF-1–ILEI with the established finding that vemurafenib regulates the tumor microenvironment, we suggest that both USF-1 and ILEI could be regulators of the tumor immune microenvironment. Future studies should seek to elucidate the role of USF-1/ILEI in this paracrine signaling context.

Additionally, USF-1 is primarily known as a regulator of glucose and lipid metabolism and is poorly described as an oncogene (48–50). In melanoma USF-1 is known as a stress responsive transcription factor involved in UV-induced, but not MITF-mediated constitutive pigmentation (51). The non-overlapping function of USF-1 with MITF in pigmentation suggests that USF-1 could play other roles in melanoma biology when MITF is poorly expressed, such as in MITF-low invasive melanoma cells. These cells are characterized by the lack of MITF but not by the presence of any major transcription factor, although ZEB1 and JUN have been proposed (20, 21, 23, 37, 38). Considering that ILEI is a regulator of MITF-low cells we speculate that USF-1 could be a novel transcription factor regulating the MITF-low invasive state (Fig. 11) (25). Future work should address the role of USF-1 in melanoma phenotype switching.

## Experimental procedures

### Animal studies

All procedures were approved by the Institutional Animal Care and Use Committee at the Medical University of South Carolina. Lung colonization experiments were conducted as follows: tail vein injection into 6–8–week-old mice were performed with 1205Lu cells expressing shSCR or shILEI 3 (1 × 10^5^ cells in 100 μl of PBS). Cells were previously characterized (25). After 8 weeks, mice were sacrificed, and lungs were harvested. Organs were formalin-fixed and paraffin-embedded. Histopathological analysis was conducted by Hollings Cancer Center Biorepository & Tissue Analysis Shared Resource. Lung colonies were counted by two individuals.

### Constructs

ILEI reporter constructs were cloned as follows: the ILEI promoter sequence from 2,300 bp upstream of the predicted transcription start site to 80 bp upstream was amplified by PCR from 501-Mel genomic DNA with 5′ KpnI and 3′ NheI primers and ligated into pBV-Luc (pBV-Luc vector (a gift from Bert Vogelstein (Addgene plasmid number 16539)) (35). Primers are listed in Table 1. Subsequent ILEI promoter truncation constructs were cloned by PCR using an alternate 5′ KpnI primer and the same 3′ NheI primer, or using a Q5 SDM kit from New England Biolabs (Ipswitch, MA). The ILEI 3′ UTR construct from stop codon to 1,620 bp downstream of the stop codon was amplified by PCR from 501-Mel cDNA with 5′ NheI and 3′ SalI primers and ligated into pmirGLO Dual Luciferase vector (RAB14 3′ UTR WT was a gift from Curt Civin (Addgene plasmid number 61489)) (52). The following constructs were used in this study: pcDNA3-cmyc (a gift from Wafik El-Deiry (Addgene plasmid number 16011) (53), pCDNA3-HA-human MYCN (a gift from Martine Roussel (Addgene plasmid number 74163) (54), pMXs-Hu-l-Myc (a gift from Shinya Yamanaka (Addgene plasmid number 26022) (55), pEGFP-N1-TFEB (a gift from Shawn Ferguson (Addgene plasmid number 38119) (56), pEGFP-N1-TFE3 (a gift from Shawn Ferguson (Addgene plasmid number 38120) (56), pCMV-Tag4A-MITF-M (wt) (a gift from Yardena Samuels (Addgene plasmid number 31151)), and USF1, CREB3L2, and ID2 coding sequence constructs in pLX304 were generated by David Root and supplied by DNASU (57–60). Corresponding empty vector for human l-MYC was constructed by digesting the l-MYC plasmid with NotI to remove the l-MYC coding sequence and religating using T4 ligase.

**Table 1:** **Primer sequences**

Primer name	Sequence
Cloning −150 KpnI ILEI promoter F	GGC GGT ACC AAG TTC AGA TTG TGC AGC G
Cloning −204 KpnI ILEI promoter F	GGC GGT ACC ACG TGG CAA GTT CAG ATT
Cloning −221 KpnI ILEI promoter F	GGC GGT ACC CAT TTT TCT CCC TCC CGT AGI
Cloning −300 KpnI ILEI promoter F	GGC GGT ACC ATG GGAT GGG TCA TTT AAA ATG TTC TGC
Cloning −2303 KpnI ILEI promoter F	GGC GGT ACC GGA TTC TCC AAA TAC TCC ATC AGT G
Cloning −80 NheI ILEI promoter R	TTA GCT AGC AAG GGC CGG AGA GCG GA
SDM E-box ILEI promoter F	CGG CAG CTC CCA AAT GGC AAG TTC AGA TTG TGC AGC GCC TGG C
SDM E-box ILEI promoter R	CCG CCG CAG CGG CCC TGC
SDM Inr ILEI promoter F	GTA AAA AAA GTG CAG CGC CTG GCC GGG
SDM Inr ILEI promoter R	TTG CTT TTT TGG AGC TGC CGC CGC CGC A
Cloning NheI ILEI 3′ UTR F	ATT GCT AGC TGG AAA TGT GGA GAG AAT TGA AG
Cloning ILEI 3′ UTR SalI R	TTA GTC GAC CTG CAA CAT TTA TTT CAC AAT CCC T
RT-PCR ACTB F	ATG CTT CTA GGC GGA CTA TG
RT-PCR ACTB R	ACA AAT AAA GCC ATG CCA AT
RT-PCR FAM3C F	GCA ACC AAA CTC AAT GAT GA
RT-PCR FAM3C R	ACC ACA GAA GAC CCA GTT GT
RT-PCR FAM3C Intron F	TTG CCCTAA TGC AGATCA TA
RT-PCR FAM3C Intron R	CAA CAA AGA AAC CCA CAA CA
RT-PCR GAPDH F	CTC CTC ACA GTT GCC ATG TA
RT-PCR GAPDH R	GGT TGA GCA CAG GGT ACT TT
RT-PCR TYR F	ACC TCT CAT TTG CAA GGT CAA A
RT-PCR TYR R	AG AGG AAC CTC TGC CTG AAA GC
RT-PCR USF1 F	GCA CTG GTC AAT TCT TTG TG
RT-PCR USF1 R	TTC TGA CTT CGG GGA ATA AG

### Cell culture conditions

The following human melanoma cell lines were used: WM3918, 501-Mel, Sk-Mel-28, WM983B, 1205Lu, and WM9. These cell lines were purchased from ATCC, Coriell, or were a generous gift from Dr. J. Alan Diehl or Dr. Alain Mauviel. All melanoma cell lines were cultured at 37 °C, 5% CO_2_ in RPMI 1640 medium (HyClone, Logan, UT) supplemented with 10% FBS (Atlanta Biologicals, Flowery Branch, GA), antibiotic–antimycotic (×100; ThermoFisher, Waltham, MA), and prophylactic plasmocin (InvivoGen, San Diego, CA) at 37 °C and in 5% CO_2_. The HMLE human mammary cell line was obtained from Dr. Sendurai Mani and cultured in Dulbecco's modified Eagle's medium/F-12 supplemented with 5% calf serum, 0.5 μg/ml of hydrocortisone, 10 μg/ml of insulin, 20 ng/ml of EGF, and 1% antibiotic–antimycotic.

Stable cell lines were generated by lentiviral transduction with Polybrene (8 μg/ml; Sigma). 24 h post-transduction the medium was changed, and 48 h post-transduction the cells were selected and cultured with 0.125–0.5 μg/ml of puromycin (InvivoGen). Pools of stably transduced cells were analyzed. shRNA sequences are listed in Table 2. UV treatments were conducted using Fisher Scientific UV Cross-linker FB-UVXL-1000 at 0, 5, or 50 J/m^2^ in room air, and then incubating in normal cell culture conditions for 24 h.

**Table 2:** **shRNA sequences**

shRNA name	Target	Sequence
pLKO.1-puro Non-mammalian shRNA	Scrambled	CCGGCAACAAGATGAAGAGCACCAACTCGAGTTGGTGCTCTTCATCTTGTTGTTTTT
TRC2-pLKO-puro TRCN000020679	USF1	CCGGGCTGGATACTGGACACACTAACTCGAGTTAGTGTGTCCAGTATCCAGCTTTTT
TRC2-pLKO-puro TRCN000020681	USF1	CCGGCACTGGTCAATTCTTTGTGATCTCGAGATCACAAAGAATTGACCAGTGTTTTT

### Lentivirus

Lentiviral shRNAs were obtained from the MUSC Hollings Cancer Center shRNA Shared Resource Technology. All vectors used in this study are listed in Table 2. Lentivirus was generated by seeding 293T (1,000,000 cells; Takara Bio, Mountainview, CA) to a 60-mm cell culture dish, and transfecting with 6 μl of Lipofectamine 2000 (ThermoFisher), 1 μg of pLKO vector, 0.75 μg of psPAX2, and 0.25 μg of pMD2.G. 24 h post-transfection the media was changed, and 48 and 72 h post-transfection the media was harvested. Viral supernatant was cleared by centrifugation, filtered through a 0.22-μm filter, and stored at −80 °C until use.

### Luciferase analysis

Cell lines were seeded at 50,000–75,000 cells per well in 24-well plate in 0.5 ml of complete medium. At 24 h the cells were transfected using X-tremeGENE 9 (Roche Applied Science, Switzerland; 100 ng of firefly experimental luciferase, 5 ng of *Renilla* control luciferase, 0.3 μl of X-tremeGENE reagent, in 10 μl of Opti-MEM, 200 ng of experimental plasmid, where indicated (*i.e.* USF1 overexpression construct), 0.6 μl of X-tremeGENE reagent, in 20 μl of Opti-MEM). At this time the cells were treated with DMSO vehicle or 1 μm vemurafenib. At 24 h post-transfection the cells were harvested with passive lysis buffer and analyzed with Dual-Glo Luciferase Assay System (Promega, Madison, WI).

### Immunoblot analysis

Whole cell lysates were extracted as follows: 100 μl of Tris-Triton lysis buffer (20 mm Tris, pH 7.5, 1% Triton X-100, 10% glycerol, 137 mm NaCl, 2 mm EDTA, and Halt Protease and Phosphatase Inhibitor mixture (ThermoFisher)) was added to 6-well cell culture plates, cells were immediately scraped, incubated on ice for 30 min, and cleared by centrifugation for 20 min at 16,000 × *g*. Protein concentrations were measured with Bradford Protein Assay (Bio-Rad). For conditioned medium immunoblots, cells were serum starved in RPMI, 0% FBS overnight, medium was harvested, and precipitated using TCA/acetone. Protein samples were denatured by incubating at 95 °C for 5 min with 1× Laemmli Reducing Denaturing Sample Buffer (300 mm Tris-Cl, pH 6.8, 10% SDS, 50% glycerol, 25% β-mercaptoethanol). 1–20 μg of whole cell lysate was resolved on an 8, 10, or 12% polyacrylamide-SDS gel, and transferred onto polyvinylidene difluoride membrane. Membranes were blocked for 1 h at room temperature in 5% skim milk, TBS with 0.01% Tween 20 (TBST) and incubated overnight at 4 °C on primary antibody + 5% skim milk/TBST. The following primary antibodies were used: ILEI (ab72182; Abcam, 1:1,000; specificity for the band between 25 and 20 kDa confirmed in Ref. 25)), α-tubulin (2144; Cell Signaling, Danvers, MA; 1:10,000), *p-*ERK T202/Y204 (4370; Cell Signaling; 1:2,000), total ERK (9120; Cell Signaling; 1:1,000), USF1 (C-20; sc-229; Santa Cruz, Dallas, TX; 1:1,000), GAPDH (sc-32233; Santa Cruz; 1:10,000), and HSP90 (sc-13119; Santa Cruz; 1:10,000). After primary antibody incubation, membranes were washed 4 × 15 min in TBST and incubated for 1 h at room temperature with secondary antibody in TBST. The following secondary antibodies were used: goat anti-mouse IgG (31430; ThermoFisher; 1:10,000) and goat anti-rabbit IgG3 (31460; ThermoFisher; 1:10,000). After secondary antibody incubation, membranes were washed 4 × 15 min in TBST and detected using Luminata Forte Western HRP substrate (EMD Millipore, Darmstadt, Germany) and HyBlot CL Autoradiography Film (Denville, Holliston, MA) or CCD camera (Bio-Rad ChemiDoc System; Bio-Rad).

### PCR analysis

Total RNA was isolated using TRIzol (ThermoFisher Scientific). Reverse transcription was performed using oligo(dT) primers and Moloney- murine leukemia virus Reverse Transcriptase (New England BioLabs). Semi-quantitative PCR was conducted on 10 ng of cDNA using Maxima Hot Start PCR Master Mix (ThermoFisher Scientific). Real-time quantitative PCR was conducted using iQ SYBR Green Supermix (Bio-Rad) using CFX384 Real-Time System (Bio-Rad). Reactions were conducted on 50 to 10 ng of cDNA. Primers are listed in Table 1. Relative gene expression was calculated using RFX Manager software, and genes were normalized to GAPDH internal control.

### Chromatin immunoprecipitation (ChIP)

ChIP protocol was modified from Carey *et al.* (61). Briefly, 1.5 × 10^7^ cells were fixed in 1% formaldehyde for 10 min at room temperature and quenched in 125 mm glycine. Cells were harvested in lysis buffer (5 mm PIPES, pH 8, 85 mm KCl, 0.5% Nonidet P-40), and centrifuged for 10 min (3,000 rpm at 4 °C). Supernatant (cytosolic fraction) was removed, and the pellet was resuspended in nuclei lysis buffer (50 mm Tris, pH 8, 10 mm EDTA, 1% SDS, protease and phosphatase inhibitor tablets). Nuclear extracts were sonicated for 10 min (30 s on and 30 s off), and cleared by centrifugation (2 times, 10 min, 13,000 rpm at 4 °C). The supernatant was considered the chromatin fraction. 100 μg of chromatin samples were precleared for 2 h at 4 °C with 30 μl of slurry ChIP-Grade Protein G-Agarose Beads (9007; Cell Signaling). The resulting supernatants were incubated overnight on a rotator at 4 °C with 5 μg of control Mouse (G3A1) mAb IgG1 Isotype Control (5415; Cell Signaling) or USF1 antibody (C-20). 30 μl of slurry ChIP-Grade Protein G-Agarose Beads was added to each sample for 2 h on a rotator at 4 °C. Beads were washed 4 times in high-salt wash buffer (50 mm HEPES, pH 7.9, 500 mm NaCl, 1 mm EDTA, 0.1% SDS, 1% Triton X-100, 0.1% deoxycholate) each time incubating for 10 min on a rotator at room temperature. Beads were next washed 2 times in TE buffer each time incubating for 10 min on a rotator at room temperature. Beads were resuspended in 300 μl of elution buffer (50 mm Tris, pH 8, 10 mm EDTA, 1% SDS) + 20 μg of RNase A (R1253; ThermoFisher Scientific) and incubated on a 55 °C heat block for 2 h. 1.6 milliunits of proteinase K (P8107S; New England BioLabs) were added prior to another incubation on a 55 °C heat block for 2 h. The samples were transferred to a 65 °C heat block for overnight elution. The samples were purified in 30 μl of H_2_O using GeneJET PCR Purification Kit (K0702; ThermoFisher Scientific). PCR was conducted with primers used for *FAM3C* promoter cloning specific for human *FAM3C* promoter from 300 to 80 bp upstream of TSS, as listed in Table 1, human *TYR* promoter or human *HMOX1* promoter (15, 42).

### Biotin DNA pulldown

DNA pulldown assays were conducted with 5′-biotinylated double-stranded annealed oligonucleotides corresponding to *FAM3C* promoter from −200 to −110 upstream of the TSS. A mutant E-box construct was used in which the E-box at −162 to −156 was mutated from CACGTG to CAAATG. A 5′-biotinylated double-stranded annealed oligonucleotide of a random sequence was used as a negative control (SCR).

Nuclear extracts were isolated by harvesting cells in lysis buffer (10 mm HEPES, 1.5 mm MgCl_2_, 10 mm KCl, 0.5 mm DTT, 0.05% Nonidet P-40, pH 7.9, protease and phosphatase inhibitor tablets) and pelleting cell nuclei. Pellets were incubated for 30 min on ice in nuclei extraction buffer (5 mm HEPES, 1.5 mm MgCl_2_, 0.2 mm EDTA, 0.5 mm DTT, 26% glycerol, pH 7.9, protease and phosphatase inhibitor) and NaCl was added to a final concentration of 300 mm. Samples were homogenized by passing 20 times through a 28-guage needle. Samples were incubated on ice for 30 min and centrifuged. The supernatants were considered the nuclear extract. Nuclear extract (20 μg) was reserved as 5% input.

Preclear beads were prepared as follows: 5′-biotinylated random oligonucleotide (SCR) was bound to streptavidin-agarose beads (50 μl of slurry + 10 μg of oligo; ThermoFisher) for 1 h on a rotator at room temperature. IP beads were prepared as follows: 5′-biotinylated *FAM3C* promoter WT or E-box mutant (CACGTG to CAAATG) was bound to streptavidin-agarose beads (50 μl of slurry + 1 μg of oligo; ThermoFisher) for 1 h on a rotator at room temperature. Preclear was conducted as follows: 400 μg of nuclear extract was incubated with preclear beads for 1 h on a rotator at room temperature. The resulting sample was centrifuged and the supernatant was precleared again for a total of three preclears. The final supernatant was incubated on IP beads overnight on a rotator at 4 °C. The beads were washed in PBS, 0.1% Triton X-100 and resolved by SDS-PAGE, transferred to nitrocellulose, and probed using antibody against USF1 (C-20; sc-229; Santa Cruz; 1:1,000).

### Statistical analyses

Data are mean ± S.D. unless indicated otherwise. *p* < 0.05 by unpaired two sample Student's *t* test is considered significant. Representative experiments are repeated at least twice.

### Wound-healing assays

Cells (3 × 10^5^, 0.5 ml of complete medium) were seeded in a 24-well plate, and a 1-ml pipette tip was used to scratch the cells. Images were recorded from 0 to 24 h, and analyzed using ImageJ (National Institutes of Health, Bethesda, MD).

## Author contributions

K. N. and P. H. H. conceptualization; K. N., T. A. D., A. C. D., B. V. H., B. J. M., and B. K. M. data curation; K. N. formal analysis; K. N. and P. H. H. funding acquisition; K. N., T. A. D., A. C. D., B. V. H., B. J. M., and B. K. M. investigation; K. N. methodology; K. N. and P. H. H. writing-original draft; K. N., T. A. D., A. C. D., B. V. H., B. K. M., and P. H. H. writing-review and editing; P. H. H. supervision; P. H. H. project administration.

## Footnotes

2 The abbreviations used are:

MEK: mitogen-activated protein kinase
ERK: extracellular signal-regulated kinase kinase
bHLH: basic-helix-loop-helix
LZip: leucine zipper
MITF: micropthalmia-associated transcription factor
USF-1: upstream stimulatory factor 1
ILEI: interleukin-like EMT inducer
EMT: epithelial-to-mesenchymal transition
TSS: transcription start site
Inr: initiator sequence
TGF: transforming growth factor
qPCR: quantitative PCR
UTR: untranslated region
GAPDH: glyceraldehyde-3-phosphate dehydrogenase
EGFP: enhanced green fluorescent protein
IP: immunoprecipitation
hnRNP: heterogeneous nuclear ribonucleoprotein.
